# Encephalic Wounds Observed in 56 Cases

**Published:** 1918-09

**Authors:** 


**Symptoms, Treatment, and Prognosis of Encephalic Wounds Observed in 56 Cases.** By A. MOULONGUET, and P. LEGRAIN. A Report before the Société de Chirurgie, May 29, 1918. *La Presse Médicale,* June 13, 1918.

The authors consider only wounds penetrating the dura mater. Out of the 56 cases, they observed motor disturbances in only 10. These were more frequently hemiplegic than monoplegic, and they were associated with more or less marked sensory disturbances, such as numbness of the upper limbs. Aphasia. hemianopsy, and Jacksonian epilepsy were observed three times each, and general epilepsy once.

In 14 cases, there were, at the time of arrival, the most completely diffused syndrome — deep coma. In two cases there were observed some of the characteristics of cerebral syndrome, like asynergy and adiadococcinesia.

*Treatment*: The authors raise the question as to the advisability of suturing primarily the outer skin as recommended by Gross, Houdart, and Tanton. The authors regard the practice as dangerous, for they insist that the conditions *sine qua non* of primary suture are complete débridement and excision which in many cerebral wounds are impossible.

The authors object further to primary suture on the ground that the brain pulsation is the most effective means of expelling devitalized tissue, foreign bodies, and bone fragments, — all of which favorable activity is defeated by primary suture.

They further dispute the soundness of the statistical argument in defence of primary suture, because they believe there has not yet been a sufficiently long experience of suture of brain wounds to establish positively the superiority of this technic.

As to operative technic, the authors prefer ether or local anesthesia to novocaine, adrenaline, or chloroform. They consider that this last, by reason of its action upon the brain, paralyses the natural defense against infection.

They make a star-shape incision with three points, carefully removing all contused tissue. Bone splinters are extracted by means of forceps and curette, guided by digital exploration.

Projectiles, whatever their size and position, should be extracted primarily, for they constitute a permanent menace. Extraction under a screen used intermittently is a most advantageous procedure. They insist upon extraction by means of the wound tract except when the missile is lodged in the side of the brain opposite to that of the entrance orifice. The electro-magnetic method of extraction, the authors find, is awkward because of the complicated nature of the machinery, and it is useless when non-magnetic bodies are involved.

Unless extraction can be accomplished before infection has set in, the authors think it better to run the chance that the seat of the infection may become encysted, and so permit of delayed extraction, than to attempt primary extraction when an abscess or inflammation has been observed.

The authors do not favor the use of drains for fear that the constantly pulsating tissues become ulcerated from the friction against the material inserted.

They believe the best treatment to be a flat dressing which will favor the repair of the meninges and maintain the cerebral equilibrium. Lumbar puncture they consider useless and even dangerous except in cases in which meningeal reaction or hypertension is clearly established.

The complications of meningitis, encephalitis, and cerebral hernia were not very frequent, Out of 56 cases, meningitis was observed in 7. Two of the cases recovered. Encephalitis was more common, 12 cases. Cerebral hernia occurred in 6 cases. The authors prefer delayed suture. In 16 cases this was accomplished at the end of about three weeks, without recourse to antiseptics.

The statistics of the 56 cases were as follows:

54 operated:

31 deaths (61 o/o).

23 definite recoveries.

The high percentage of deaths was due chiefly to the seriousness of the wounds and to the time elapsed before operation.

